# Supplementary figures and images for: An enhanced therapeutic effect of repetitive transcranial magnetic stimulation combined with antibody treatment in a primate model of spinal cord injury
Source: PLoS One. 2021 Jun 2;16(6):e0252023. doi: 10.1371/journal.pone.0252023 (PMC8172028; doi:10.1371/journal.pone.0252023)

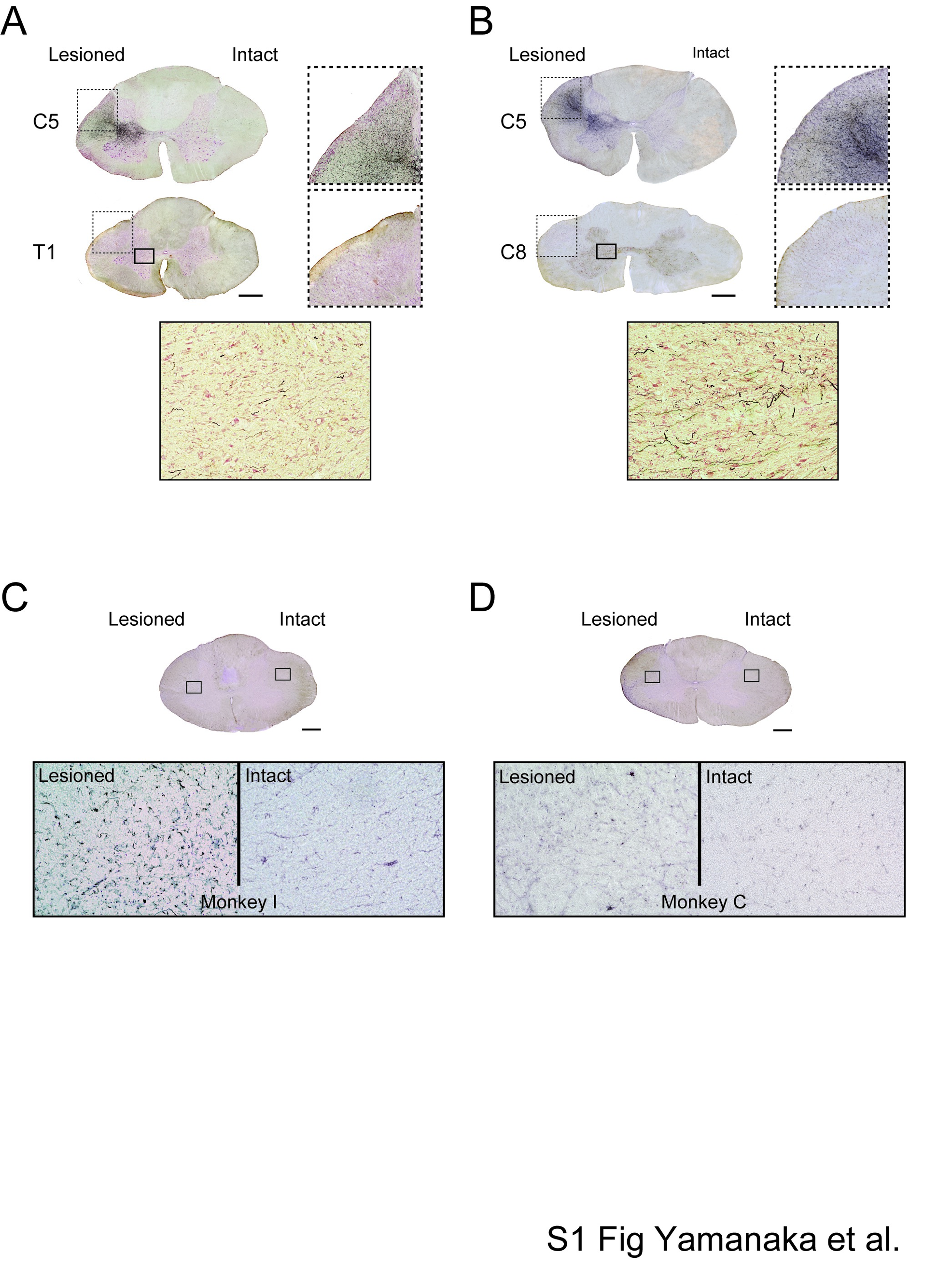

Supplement: S1 Fig — (A, B) CST fibers anterogradely labeled with BDA injected into the contralesional MI at the spinal levels above (C5) and below (C8/T1) the SCI lesion site in monkeys I (A; Control SCI) and C (B; RGMa-Ab+rTMS). Right panels are higher magnifications of the dorsolateral funiculus (dotted squares), and panels at the bottom are higher magnifications of lamina VII (rectangles) in the sections at the C8/T1 levels. (C, D) Microglial infiltration, as confirmed with Iba1 immunostaining, just below the SCI lesion site in monkeys I (C; Control SCI) and C (D; RGMa-Ab+rTMS). Lower panels are taken from the rectangles in the whole sections (upper panels). All sections were counterstained with Neutral red. Scale bars, 1 mm. (TIF) [file pone.0252023.s001.tif]
