# Supplementary material for: An enhanced therapeutic effect of repetitive transcranial magnetic stimulation combined with antibody treatment in a primate model of spinal cord injury
Source: PLoS One. 2021 Jun 2;16(6):e0252023. doi: 10.1371/journal.pone.0252023 (PMC8172028; doi:10.1371/journal.pone.0252023)
Supplement: Attachment — Submitted filename: Reply to Reviewer.docx [file pone.0252023.s002.docx]

We are grateful to the Reviewer for providing us with precious comments. Here are point-by-point responses to the comments raised by the Reviewer.

**Responses to the comments raised by the Reviewer**

Comment 1

*The cervical enlargement of the spinal cord was cut serially into 40-µm thick cross sections for Nissl staining. Surprisingly, the authors did not present any images from stained specimens. It is incomprehensible why no parallel sections were cut from such valuable samples. It would have been possible to examine glia activation and other changes by using immunohistochemistry, etc.*

Response to Comment 1

According to this comment, we have added photomicrographs of representative Nissl-stained sections (taken from four cases) in Figure 1B to show actual images of SCI lesions. As described in the Materials and methods section (Histological and statistical analyses; page 10, lines 209-211), SCI lesions were demarcated in the Nissl-stained sections and overlaid on a template section at the C6/C7 level, considering some deformation and tissue damage due to scar formation after SCI lesions.

Comment 2

*The authors repeatedly mentioned the rewiring/sprouting of corticospinal tract (CST). To detect the integrity of the main CST should have been assessed by using immunostaining for Protein kinase C gamma (PKCγ), which specifically labels the main CST. It would have been possible to examine the CST at any distances rostrally and caudally from the injury center.*

Response to Comment 2

According to this comment, we have added some data on PKCγ immunohistochemistry as S1 Fig (A, B) to identify the main CST at the levels rostral (C5) and caudal (C8) to SCI lesions. Moreover, also in relation to Comment 1, we have provided some data on Iba1 immunohistochemistry as S1 Fig (C, D) to examine the infiltration of microglial cells around SCI lesions at the C6/C7 level.

**Responses to the comment of the journal requirement**

*In order to comply with PLOS ONE's guidelines for non-human primate experiments (http://journals.plos.org/plosone/s/submission-guidelines#loc-non-human-primates), please provide additional details regarding housing conditions, feeding regimens, environmental enrichment, and all relevant steps taken to alleviate suffering (analgesia, details about humane endpoints and their application, etc.). Also indicate how often animal care staff monitored the health and well-being of the animals and the criteria used to make such assessments.*

Response to Comment

According to the journal requirement, we have added some descriptions in the corresponding part of the Materials and Methods section (page 5, lines 90-102) as follows:

The experimental protocols were approved by the Animal Welfare and Animal Care Committee of the Primate Research Institute, Kyoto University (Permission No. 2016-053). All experiments were conducted according to the Guidelines for Care and Use of Nonhuman Primates (Ver. 3, 2010) prepared by the Primate Research Institute. All monkeys used in this study, weighing less than 10 kg, were kept in indoor individual cages (0.89 m×0.63 m×0.82 m) on a 12-h on/12-h off lighting schedule. In each cage, the monkeys were given *ad libitum* access to food and water, and a chain-hung wood block as a toy. The monkeys were monitored closely, and animal welfare was assessed on a daily basis and, if necessary, several times a day. This includes veterinary examinations to make sure animals are not suffering. If animals experience pain, they receive pain medications. If pain cannot be relieved, or if veterinary examinations reveal signs of suffering that cannot be relieved by analgesics, antiemetics, or antibiotic therapy, animals are euthanized.
