# Supplementary material for: An enhanced therapeutic effect of repetitive transcranial magnetic stimulation combined with antibody treatment in a primate model of spinal cord injury
Source: PLoS One. 2021 Jun 2;16(6):e0252023. doi: 10.1371/journal.pone.0252023 (PMC8172028; doi:10.1371/journal.pone.0252023)
Supplement: Attachment — Submitted filename: Responses to Reviewers comments.docx [file pone.0252023.s003.docx]

We are grateful to the Reviewer for critical reading of our revised manuscript and providing us with further insightful comments. Here are point-by-point responses to the Reviewer’s comments.

First of all, we apologize for our misleading presentation of PKCγ immunohistochemical data in Supplementary Figure 1. For analyzing the extent of SCI lesions through identification of CST fibers, the anterograde tracer, biotinylated dextran amine (BDA), was in fact injected into the primary motor cortex contralateral to the SCI lesion site in all monkeys, Nevertheless, we totally failed to take this into consideration when the original manuscript was prepared. To visualize CST fibers anterogradely labeled with BDA, the use of the avidin-biotin-peroxidase complex is indispensable. This obviously makes it untenable to evaluate immunohistochemical data to be obtained with the same method, especially at the spinal level above the SCI lesion site. In this revised version, we therefore would like to replace the data on PKCγ immunostaining with those on BDA anterograde tract-tracing (see Supplementary Figure 1A and B), if the Reviewer might admit.

**Responses to the comments raised by the Reviewer**

Comment 1

*Figure 1. Images of representative Nissl-stained sections taken from monkeys show weak tissue integrity at the intact left side. It appears at first glance, as if the tissue underwent freezing damages, but is not clear what and how happened. It appears that the injured side contains a relatively intact scar tissue. Please explain and correct these images.*

Response to Comment 1

As pointed out by the Reviewer, Nissl-stained sections with weak tissue integrity were included in Figure 1, due to freezing damages and so on as the Reviewer assumed. Therefore, we have replaced the corresponding three sections with the fresh ones.

Comment 2

*Suppl. Fig. 1A and B. Why do these images overlap? Please indicate the injured and intact side. Please show the CST at higher magnification (insert high power images as inset, or similar) to visualize better the difference between the groups. The PKCgamma signal is stronger on the injured side than on the intact one. Please explain and amend/replace the figure.*

Response to Comment 2

As explained above, we have replaced the data on PKCγ immunostaining with those on BDA anterograde tract-tracing, reflecting the presentation manner suggested by the Reviewer (see Supplementary Figure 1A and B). According to this revision, we have also changed and/or added some related descriptions in the corresponding parts of the Materials and methods section (page 5, lines 90–91; page 9, line 203 through page 10, line 213; page 10, lines 216–217; page 11, lines 228–246), the Results section (page 13, lines 285–293), and the Discussion section (pages 16–17, lines 366–377 as well as in the legends of Figure 1 (page 5, lines 111 through page 6, 112) and Supplementary Figure 1 (page 21, line 508 through page 22, line 517).

Comment 3

*Suppl. Fig 1C and D. Please insert an overview of spinal cords in lower magnification and indicate the source of higher magnification images (with boxes of sampling).*

Response to Comment 3

According to the Reviewer’s advice, we have added spinal sections in lower magnification and indicated the source of higher magnification images (see Supplementary Figure 1C and D).

Comment 4

*Furthermore, authors need to indicate the staining in the Fig.1 and Suppl. Fig.1., too.*

Response to Comment 4

According to the Reviewer’s suggestion, we have added some descriptions in the legends of Figure 1 (page 6, lines 120–121) and Supplementary Figure 1 (page 22, lines 516–517).

Comment 5

*In results section the glia and CST results should be better explored. At present it is just one sentence. Please explicitly discuss them in the discussion section.*

Response to Comment 5

As described in Response to Comment 2, we have thoroughly revised the corresponding parts of the Results section (page 13, lines 285–293) and the Discussion section (pages 16–17, lines 366–377).
